# Characterization of NGF, trkA^**NGFR**^, and p75^**NTR**^ in Retina of Mice Lacking Reelin Glycoprotein

**DOI:** 10.1155/2014/725928

**Published:** 2014-01-30

**Authors:** Bijorn Omar Balzamino, Filippo Biamonte, Graziana Esposito, Ramona Marino, Francesca Fanelli, Flavio Keller, Alessandra Micera

**Affiliations:** ^1^IRCCS-G.B. Bietti Foundation, Rome, Italy; ^2^Institute of Histology and Embryology, School of Medicine “A. Gemelli”, Catholic University of the Sacred Heart, Rome, Italy; ^3^Laboratory of Developmental Neuroscience and Neural Plasticity, University Campus Bio-Medico, Rome, Italy; ^4^Laboratory of Ophthalmology, IRCCS-G.B. Bietti Foundation, Via Alvaro del Portillo 21, 00128 Rome, Italy

## Abstract

Both Reelin and Nerve Growth Factor (NGF) exert crucial roles in retinal development. Retinogenesis is severely impaired in *E-reeler* mice, a model of Reelin deficiency showing specific Green Fluorescent Protein expression in Rod Bipolar Cells (RBCs). Since no data are available on Reelin and NGF cross-talk, NGF and trkA^NGFR^/ p75^NTR^ expression was investigated in retinas from *E-reeler* versus control mice, by confocal microscopy, Western blotting, and real time PCR analysis. A scattered increase of NGF protein was observed in the Ganglion Cell Layer and more pronounced in the Inner Nuclear Layer (INL). A selective increase of p75^NTR^ was detected in most of RBCs and in other cell subtypes of INL. On the contrary, a slight trend towards a decrease was detected for trkA^NGFR^, albeit not significant. Confocal data were validated by Western blot and real time PCR. Finally, the decreased trkA^NGFR^/ p75^NTR^ ratio, representative of p75^NTR^ increase, significantly correlated with *E-reeler* versus E-control. These data indicate that NGF-trkA^NGFR^/ p75^NTR^ is affected in *E-reeler* retina and that p75^NTR^ might represent the main NGF receptor involved in the process. This first NGF-trkA^NGFR^/ p75^NTR^ characterization suggests that *E-reeler* might be suitable for exploring Reelin-NGF cross-talk, representing an additional information source in those pathologies characterized by retinal degeneration.

## 1. Introduction

Reelin and Nerve Growth Factor (NGF) take part in retinogenesis, and their increased levels occur in inflamed/degenerating retina [1–3]. Reelin is a highly conserved extracellular glycoprotein, released by neurons/accessory cells and signals via specific surface receptors belonging to the Apolipoprotein E and the very low density lipoprotein families (ApoER2 and VLDLR) and via adaptor protein Dab1 [4, 5]. Reelin expression peaks during retinogenesis, allowing migration/positioning and differentiation of retinal cells (physiological upregulation), returns to baseline levels in adulthood (physiological downregulation), and increases again following local injury/degeneration (pathological upregulation) [6, 7]. Reelin deprivation causes a macroscopic modification of the retinal structure, with incorrect cells distribution and synaptic circuitry alteration, including a decrease in Rod Bipolar Cells (RBCs) density and an abnormal distribution of their processes in the Inner Nuclear Layer (INL), as well described in the *reeler* model [1, 8].

In the visual system, NGF exerts pleiotropic effects during development and guarantees homeostasis during adulthood [2, 3, 9–11]. NGF appears to exert multiple effects in both the neurons and accessory cells (proliferation, migration, differentiation, cytoskeletal reorganization, survival, apoptosis, etc.) [2, 3, 11]. NGF plays a crucial role during retinogenesis, influencing neuritic outgrowth, survival, and apoptosis, together with other neurotrophins and their related receptors, while in adulthood NGF is involved in several pathophysiological processes (homeostasis, ischemia, glaucoma, etc.) [2, 3, 10, 11]. NGF is produced and used in an autocrine/paracrine fashion by Retinal Ganglion Cells (RGCs), Bipolar and other retinal cell types (horizontal and amacrine cells, Müller glia) [11]. The biological effects of NGF are directly dependent on the specific binding to two different cell surface receptors, the tyrosine kinase trkA^NGFR^ and the glycoprotein p75^NTR^ [12, 13]. NGF/trkA^NGFR^ promotes the survival and recovery of RGCs, as observed in experimental models and after intraocular injection of NGF [2, 11, 14, 15]. The crucial contribution of the trkA^NGFR^/p75^NTR^ ratio in the survival of RGCs has been recently envisaged [13, 15]. Previous studies showed an impaired NGF expression in Reelin-deficient mice [16, 17].

In view of all these findings a possible cross-talk between NGF and Reelin during retinal development might be hypothesized, suggesting an abnormal NGF pathway in Reelin-deficient retinas. To address this question, a homozygous *reeler* mice model was developed from founder couples and used to investigate both the biochemical and molecular expression of NGF-trkA^NGFR^/p75^NTR^.

## 2. Materials and Methods

### 2.1. Ethics Statement

All experiments were performed in compliance with the ARVO Statement for the Use of Animals in Ophthalmic and Vision Research. Animal care procedures were conducted in conformity with the Intramural Committee and Institutional guidelines, in accordance with national and international laws and policies (EEC Council Directive 86/609, OJ L 358, 1, December 12, 1987; NIH Guide for the Care and Use of Laboratory Animals, NIH Publication 85-23, 1985). The experimental protocol was approved by the Ethical Committee of “Tor Vergata” University (Rome, Italy).

### 2.2. Animals and Genotyping

Founder couples of *reeler* strain (B6C3Fe-a/a-rl; Jackson Laboratories, Bar Harbor, ME, USA), carrying the rl^jx−/−^ mutation in a C57BL/6J background, were purchased from Charles River (Calco, Italy). The colony was housed at animal house facility (“Tor Vergata” University) under standard conditions (12 hrs light/dark cycle, temperature 21 ± 1°C, and relative humidity 60 ± 10%). Both water and food were freely available (Enriched Standard Diet, Mucedola, Settimo Milanese, Italy). To obtain the double-mutant* reeler*-L7-EGFP strain (referred to as *E-reeler* in this study), B6C3Fe-a/a-rl and B6-FVB-Tg (Pcp2-EGFP)2Yuza/J strains (Jackson Laboratories) were crossed and then backcrossed, according to a standard procedure [18]. The offspring from the 8th generation were used as donors that were hemizygotes for the Green Fluorescent Protein (GFP) locus. The B6C3Fe-L7-EGFP mice were used as control (wild-type rl^jx+/+^ genotype; E-control). Since GFP expression is under control of the L7 promoter, high levels of this live cell marker (lacking toxicity) characterize RBCs and allow their easy analysis at confocal microscopy (resistant to bleaching) [19].

For the present study, a total of *n* = 62 eyes (31 mice) were used and grouped as follows: *n* = 32* E-reeler* eyes (21 ± 1 days; body-weight range: 9–11 g) and *n* = 30 E-control eyes (21 ± 1 days; body-weight range: 12–14 g). In a pilot study, RBCs were quantified at P7, P14, P21, and P35, in order to choose the best time-point for analyses (*n* = 3/time-point; *n* = 9  *E-reeler,* and *n* = 3 E-control).

EGFP expression and *reeler* genotype were assessed as previously reported, with minor modifications [20, 21]. Briefly, 1 *μ*g DNA was extracted from tails and placed in a 10x reaction buffer (100 mM Tris-HCl, pH 8.3, 500 mM KCl, and 15 mM MgCl_2_) containing 10 mM dNTPs, 20 *μ*M random primers, and 5 U/mL recombinant Taq polymerase (Euroclone, Milan, Italy). DNA amplification was performed in a PTC-100 cycler (MJ Research Inc., Watertown, MA). Specific primers for GFP detection and genotyping studies were synthesized by Invitrogen (Grand Island, USA). Detailed primer sequences, gene accession number and amplification profiles are reported in Table 1(A). DNA products and ladder (100 bps molecular marker; Takara Bio Inc., Shiga, Japan) were separated in 0.8–1.5% agarose gel containing ethidium bromide (0.5 *μ*g/mL; Appligene Oncor). Gels were acquired by the Kodak EDAS 290 imaging system (Kodak, Tokyo, Japan).

### 2.3. Tissue Sampling

*E-reeler* and E-control mice were deeply anaesthetized by 2 mg/mL ketamine (0.2 mL/10 gr body-weight; Ketavet, Gellini Farmaceutici, Italy) and 0.23 mg/mL medetomidine (0.24 mL/10 gr body-weight; Domitor, Orion Corp., Espoo, Finland) intraperitoneal injection.

For confocal microscopy analysis, mice were perfused through the left ventricle with saline solution (3 min), followed by 4% buffered paraformaldehyde (PFA; 5 min). Enucleated eyes (*n* = 10 eyes for *E-reeler* and *n* = 8 eyes for E-control) were postfixed in the same fixative (48 hrs) and cryoprotected in 10% sucrose (24 hrs).

For molecular and biochemical analysis, mice were sacrificed by cervical dislocation and the retinas were quickly processed for biochemical (*n* = 8 eyes each, for both *E-reeler* and E-control) or molecular (*n* = 14 eyes each, for both *E-reeler* and E-control) analysis. The dissection of retinas from fresh enucleated eyes was carried out under a dissector microscope (SMZ645; Nikon, Tokyo, Japan), equipped with cold-light optic fibers (PL2000 photonic; Axon, Vienna, Austria).

### 2.4. Immunofluorescence and Digital Analysis

Postfixed and cryoprotected eyes were quickly frozen in dry ice, embedded in OCT medium (TissueTek; Leica, Heidelberg, Germany), and sectioned (CM3050 cryostat; Leica Microsystems, Rijswijk, The Netherlands). Serial sections (7 *μ*m) were placed (*n* = 3 sections/slide, *n* = 6 slides/retina) onto gelatinized slides, preheated to increase tissue attachment, and stored at −20°C. Both antigen retrieval (0.05% trypsin-EDTA solution, 2 min) and blocking/permeabilizing (1% BSA and 0.5% Triton X100 in PBS, 15 min) steps were performed before the addition of specific antibodies: anti-NGF (sc-549), anti-trkA^NGFR^ (sc-118), and anti-p75^NTR^ (sc-6188) antibodies, all from Santa Cruz Biotech (Santa Cruz, CA). A quenching passage was also carried out to minimize PFA background and slightly reduce the higher fluorescent expression of the E-construct. Cy3-conjugated donkey species-specific antibodies (1 : 500–700; Jackson ImmunoResearch Europe Ltd., Suffolk, UK) were used to bind all primary antibodies. Nuclear counterstaining was performed with TOTO3-Iodide (Molecular Probes, Eugene, OR) while GFP expression specifically identified RBCs [19]. Isotypes (negative controls) were carried out in parallel with the omission of primary antibodies and used for appropriate channel series acquisition and related background subtractions. Slides were coverslipped using a glycerol gelatin mounting medium (Sigma-Aldrich, St. Louis, CA). Serial images were acquired by C1 software connected to an inverted microscope (Eclipse TE2000U, Nikon). Digital images (pixel size: 512 × 512 or 1024 × 1024 dpi) were saved, converted into 8-bit TIFF images, and subjected to densitometric analysis (Image J v1.43; NIH-http://rsb.info.nih.gov/ij/). Single integrated optical density (IntDen) was registered for *E-reeler* and E-control retina (*n* = 5 optic fields/slide/retina; ×40/dry 0.75 DIC M/N2), in terms of specific RBCs density and dendrite length (*n* = 5 sections/animal). IntDen data were collected: mean values (±SD) were calculated and subjected to statistical analysis. Quantification of RBCs in retinas was performed by evaluating the number of the GFP-expressing RBCs in the INL. A grid of 27 × 18 fields (field size: 25 *μ*m × 27.5 *μ*m) was printed onto a transparent sheet and attached to the screen of a 15′ LCD-display. Three representative regions were analysed as follows: left, right, and across the optic nerve over five consecutive slides/mice/experimental group. Cells outside the region of interest were automatically ignored and the results were averaged as a percentage of positive cells. All counts were performed under blind conditions and presented as mean ± SD.

### 2.5. Western Blot

Dissected retinas were homogenized in 70 *μ*L modified RIPA Buffer (50 mM Tris-HCl, 150 mM NaCl, 1% Triton-X100, 5 mM EDTA, 100 mM NaF, and 1 mM PMSF; pH 7.5) and briefly sonicated to shrink DNA (VibraCell equipped with microtip; Sonics & Materials, Inc., Newtown, USA) [22]. Total proteins were quantified with the DC protein assay kit (BioRad Laboratories Inc., Hercules, CA), by using the A1000 Spectrophotometer (Nanodrop, Celbio, Milan, Italy). Normalized samples (50 *μ*g) were subjected to 7.5% or 12% SDS-PAGE, under reducing conditions (130 V/frontline; Miniprotean apparatus, Biorad). Electrophoresed proteins were transferred onto Hy-Bond membranes (GE Healthcare, Buckinghamshire, UK), in the presence of 48 mM Tris-Cl (pH 6.8), 39 mM glycine, 0.0037% SDS, and 20% methanol solution (10 V/55 min; semidry condition, Transblotting apparatus, Biorad). Membranes were stained with 0.5% Ponceau S in acetic acid (ICN, Milan, Italy) to verify protein transfer, washed twice in 0.5% Triton X100-Tris buffered saline (TBS: 20 mM Tris-HCl and 150 m NaCl, pH 7.5) for 30 min, and blocked in 0.05% Tween-20 TBS (TW-TBS) containing 5% nonfat dry milk for 1 hr. The membranes were then probed (4°C/18 hrs) with the following primary antibodies: anti-NGF (sc-549), anti-trkA^NGFR^ (sc-118), anti-p75^NTR^ (sc-6188) from Santa Cruz, and anti-Actin (ab-3280) from Abcam (Cambridge, UK). Membranes were washed in TW-TBS and subsequently incubated with POD-conjugated donkey species-specific antibodies (1 : 30000; Jackson ImmunoResearch) for 90 min. Detection of specific signal was performed by using an enhanced Chemi-Luminescent system (West Femto Sensitivity Substrate; Pierce, Rockford, IL). Alternatively, membranes were exposed to Hybond filters in appropriate cassette and developed in appropriate solutions. Both membranes and filters were acquired by 1D Image Station (Kodak). Data were saved as 8-bit TIFF files and exported to be shown after Adobe Photoshop CS3 assembly (Adobe System, San Jose, CA). Densitometric analysis was performed using the 1D Image software (Kodak) and related Optical Density (OD) values, referred to as normalized samples, were shown in bar plots.

### 2.6. Real Time PCR Amplification

Dissected retinas were pretreated with Proteinase K (20 mg/mL, 56°C/3 hrs; Finnzyme, Milan, Italy) in modified HIRT buffer (50 mM Tris-Cl, pH 8, 1 mM EDTA, 1% Tween20). Total RNA was extracted 1 : 1 with TRIfast, according to a standard procedure (EuroClone), and resuspended in 10 *μ*L fresh RNase free water (Direct Q5, Millipore Corporation, Billerica, MA). To eliminate any genomic DNA contamination, all total RNA samples were treated with RNase-free DNaseI, according to the supplier's protocol (2 U/*μ*L; AM-1907; Turbo DNA free kit; Ambion Ltd., Huntingdon, Cambridgeshire, UK). Total RNA samples were checked for RNA quantity/purity (>1.8; A280 program, Nanodrop) and for absence of RNA degradation (1% agarose gel analysis). Equivalent amounts of RNA (1 *μ*g) were used as template to generate cDNAs, according to the IMPROM manufacturer's procedure (Promega Corp., Madison, USA), in a one cycler programmable thermocycler (PeqLab Biotech, Erlangen, Germany). The resulting cDNAs were amplified using the SYBR Green PCR core reagent kit (Applied Biosystems, Foster City, CA) in an Opticon2 programmable thermocycler (MJ Research), according to standard procedures [23]. Samples were amplified in duplicate and in parallel with negative controls (either without template or with mRNA as template). Real cycle thresholds (Cts) were recorded during linear amplification and normalized to those of referring genes run in parallel (nCts = Ct_target_ − Ct_referring_). Averages were calculated from these replicates and expressed as normalized Ct or as expression ratio of a normalized target gene (fold changes in log2-scale), according to REST^©^ analysis [24]. Both primer sequence and amplification profile are accurately reported in Table 1(B). The specific primers were designed using Primer3 software (http://www.primer3.com) and synthesized by MWG Biotech (Ebersberg, Germany). GenBank software was used to select the complete mRNA sequence of each gene investigated (http://www.ncbi.nlm.nih.gov/ Genbank; provided by the National Center for Biotechnology Information, Bethesda, MD). Primer specificities were confirmed by single melting curves, monitored during amplification. In random tests, PCR products were separated on 2.5% agarose gel and acquired by 1D Kodak software to verify the presence of a single amplicon.

### 2.7. Statistical Analysis

All data are shown as mean ± SD (in text) and mean ± SEM (in bar plots). Parametric ANOVA analysis followed by a Tukey-Kramer post hoc comparison was used to estimate differences between groups [25]. The statistical package used was StatView II for PC (Abacus Concepts Inc., Barkley, CA). REST/ANOVA coupled analysis was carried out for molecular comparisons. trkA^NGFR^/p75^NTR^ ratio was calculated according to the single Cts values recorded during linear amplification and normalized to those of referring genes run in parallel (nCts), where Cts are inversely proportional to mRNA expression [22]. Kendall's rank coefficient (Tau) was also calculated to identify correlation between GFP versus p75^NTR^ and trkA^NGFR^ versus p75^NTR^. A probability of *P* < 0.05 was presumed to reflect statistical significant difference between groups.

## 3. Results

**Figure 1:**
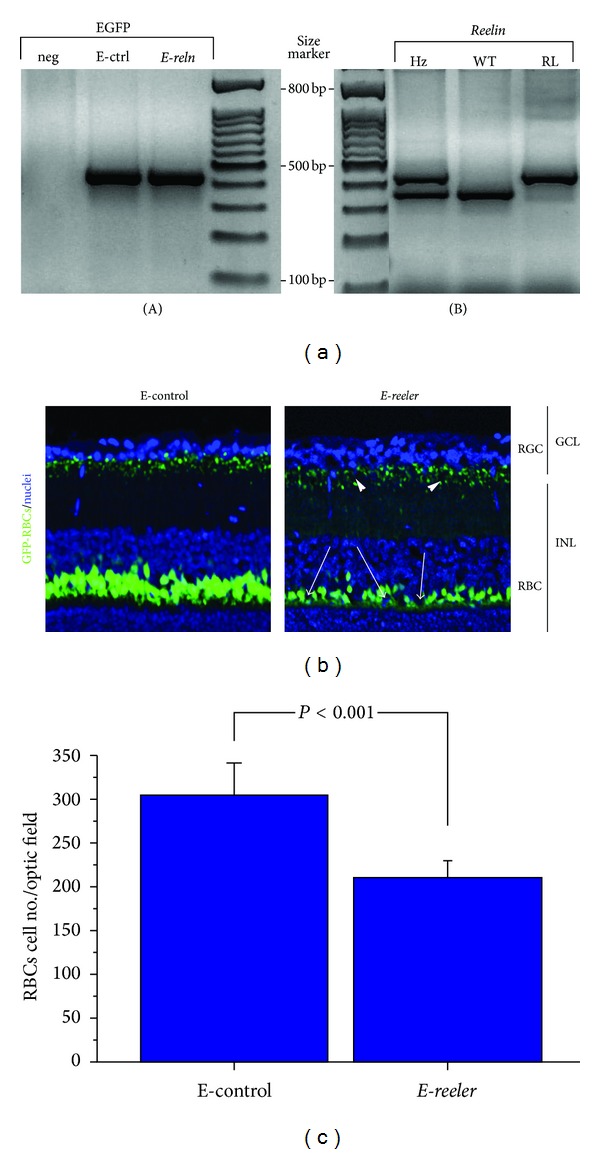
*E-reeler* model. (a) Agarose gel representative of EGFP and *Reelin* gene amplification. (A) EGFP expression in E-control (E-ctrl) and *E-reeler* (*E-reln*) tail genome, with respect to negative control (neg), not expressing GFP linked to L7-EGFP construct; (B) Reelin expression in control (WT), heterozygote (Hz), and *reeler *(Rl). The higher band (380 bps) represents *reeler* status while the lower one (280 bps) represents control rank. (b) Representative confocal microscopy image showing GFP-expressing RBCs (green) and nuclei stained with TOTO3-Iodide (blue). A decrease in the number of RBCs populating the INL is visible in *E-reeler* retina (arrows), as compared to E-control counterpart. Note the reduction of GFP-fluorescence of both the dendrite length and synapses (GFP staining) indicated by arrowheads in the *E-reeler* retinas (×400). (c) Number of GFP-bearing RBCs in *E-reeler* retinas, compared to the E-controls. Note the significant decrease of fluorescent cells (*P* < 0.001). Abbreviations: GFP, Green Fluorescent Protein; RGCs, Retinal Ganglion Cells; INL, Inner Nuclear Layer; RBCs, Rod Bipolar Cells.

A preliminary observation of *E-reeler* mice was carried out between P7 and P35 showing ataxia, eating complications (typical of *reeler* mice), and survival difficulties for the majority of animals, in the absence of appropriate handling [1]. Accordingly, the P21 time-point was selected for the following studies of characterization, and only *E-reeler* mice carrying both the *reeler* mutation and EGFP expression were included in the study. The genotyping is shown in Figure 1(a): as depicted in (A), a 500 bp band was observed in *E-reeler* and E-control DNA extracts, as compared to a negative control, confirming the positive EGFP genotyping; as depicted in (B), two DNA fragments were observed in DNA extracts: one corresponding to the wild-type allele (280 bp; WT) and the other to the *reelin* allele (380 bp; Rl). Both bands are visible in the heterozygous mouse, not used in these studies.

According to Oberdick and coworkers procedure, both *E-reeler* and E-control retinas express GFP-specific fluorescence mainly localized in the dendrites, soma, and axon terminals of RBCs, allowing their easy recognition with confocal microscopy [20]. As shown in Figure 1(b) (merge), a decrease in the number (arrows) and dendrite length of RBCs populating the INL, as well as their synaptic buttons (arrowheads) in the GCL, was observed in *E-reeler* retinas. Digital analysis carried out on serial images showed a 27.2% decrease in GFP immunoreactivity in *E-reeler* retina (20899 ± 4663 versus 28727 ± 8134 IntDen, resp., *E-reeler *versus E-control; *P* < 0.001). GFP-bearing RBCs were also counted in serial sections, showing a 27.8% decrease in the *E-reeler* retinas as compared to E-control ones (resp., 209 ± 4 versus 287 ± 81 cells/optic field; *P* < 0.001; Figure 1(c)).

### 3.1. NGF Expression

**Figure 2:**
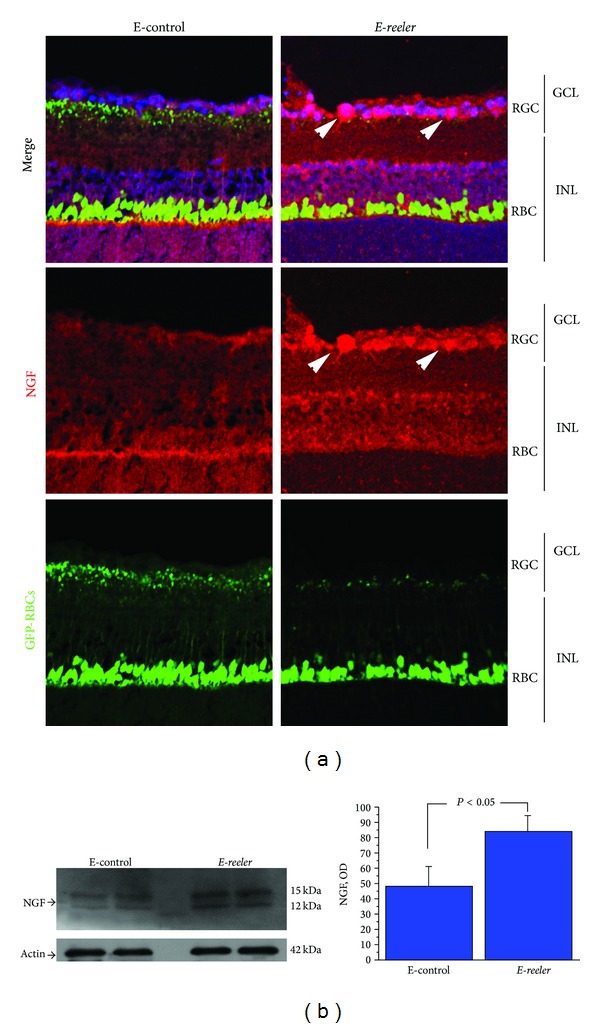
Expression of NGF in *E-reeler* retina. (a) Confocal microscopy showing images of GFP-expressing RBCs (green), NGF immunoreactivity (red), and nuclear staining (blue). As indicated by arrowheads, in the merge and NGF single staining, INL cells strongly immunoreacted with the NGF antibody. Some immunoreactivity was also observed in other structural and accessory cells (×400). (b) Representative 12% SDS-PAGE and relative densitometric analysis of E-control and *E-reeler* retinal extracts probed with the NGF antibody (OD values; *P* < 0.05). The size-marker was run between the two groups. Abbreviations: GCL, Ganglion Cell Layer; INL, Inner Nuclear Layer; RGCs, Retinal Ganglion Cells; RBCs, Rod Bipolar Cells; GFP, Green Fluorescent Protein; OD, Optical Density.

In order to understand whether NGF is affected in *E-reeler* retina, serial sections were probed with specific NGF antibody and NGF immunoreactivity was evaluated in the INL and GCL. As shown in Figure 2(a), NGF immunoreactivity was increased in RBCs, as well as in both GCL and other INL cells (arrowheads) of *E-reeler* retina. Therefore, the digital analysis carried out on these sections showed a 13.9% increase of NGF immunofluorescence in *E-reeler* retinas (32705 ± 2197 versus 27756 ± 1124 IntDen, resp., *E-reeler* versus E-control; *P* < 0.05). In line with this result, Western blot and the related OD measurements showed a 37.4% NGF increase (both 12 and 15 kDa bands) in the *E-reeler* (*P* < 0.05; Figure 2(b)). Finally, the molecular analysis of *E-reeler* mRNA extracts did not show a significant effect on NGF mRNA expression (0.39_2log⁡_ ratio; 7.21 ± 1.05 versus 7.43 ± 1.89 nCts, resp., *E-reeler* versus E-control; *P* > 0.05).

### 3.2. trkA^NGFR^ Expression

**Figure 3:**
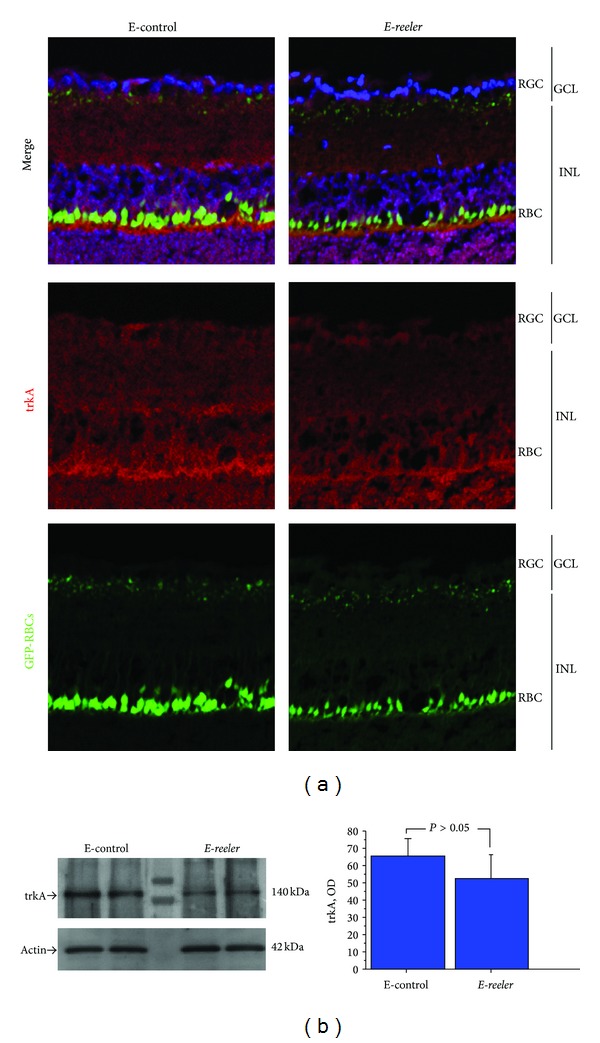
Expression of trkA^NGFR^ in *E-reeler* retina. (a) Confocal microscopy showing images of GFP-expressing RBCs (green), trkA^NGFR^ immunoreactivity (red), and nuclear staining (blue). A weak trkA^NGFR^ immunoreactivity was observed at the RBCs (body and dendrites). trkA^NGFR^ staining was less intense across the INL and in some cells inside the GCL of the *E-reeler* retina (×400). (b) Representative 7.5% SDS-PAGE and relative densitometric analysis of E-control and *E-reeler* retinal extracts probed with the trkA^NGFR^ antibody (OD values; *P* > 0.05). The size-marker was run between the two groups. Abbreviations: GFP, Green Fluorescent Protein; RGC, Retinal Ganglion Cells; GCL, Ganglion Cell Layer; INL, Inner Nuclear Layer; RBCs, Rod Bipolar Cells; OD, Optical Density.

Since trkA^NGFR^ is widely considered the main NGF receptor involved in cell migration, proliferation, and differentiation, trkA^NGFR^ protein expression was investigated by probing both* E-reeler* and E-control retinas. As shown in Figure 3(a), trkA^NGFR^ immunoreactivity was detected in both the INL and GCL of control retinas (merge). A trend towards a 5.64% decrease of trkA^NGFR^ immunoreactivity was quantified in the RGCs of* E-reeler* retinas (22601 ± 2391 versus 23953 ± 1278 IntDen, resp., *E-reeler* versus E-control; *P* > 0.05). Accordingly, Western blot and OD analysis did not show any significant trkA^NGFR^ differences between the *E-reeler* versus the E-control protein extracts (Figure 3(b)). These results were corroborated by molecular analysis (−0.14_2log⁡_ ratio; 17.04 ± 1.81 versus 16.63 ± 0.54 nCts, resp., *E-reeler* versus E-control; *P* > 0.05).

### 3.3. p75^NTR^ Expression

**Figure 4:**
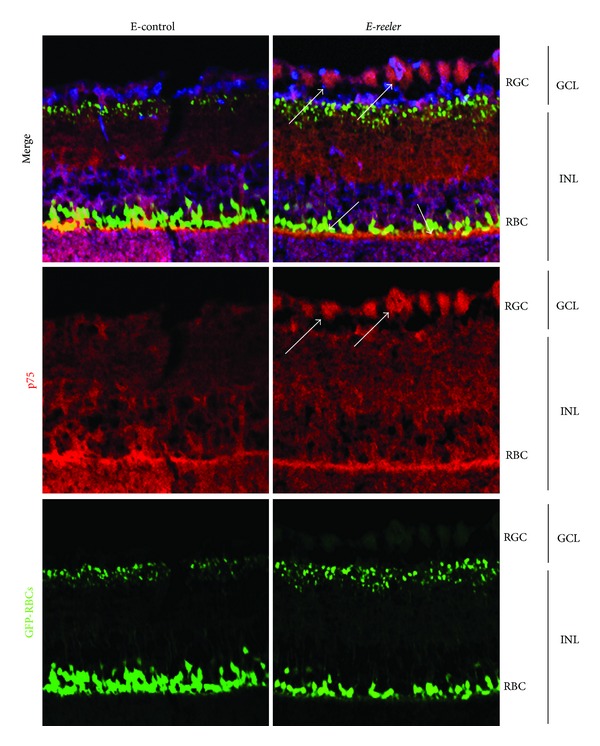
p75^NTR^ localization in *E-reeler* retina. Confocal microscopy showing images of GFP-expressing RBCs (green), p75^NTR^ immunoreactivity (red), and nuclear staining (blue). Arrows point to a noticeable staining of RGCs, RBCs, and accessories/glial cells localized in the GCL/INL of *E-reeler* retina. Note the intense cytoplasmatic p75^NTR^ localization in both RGCs and RBCs. Abbreviations: GFP, Green Fluorescet Protein; GCL, Ganglion Cell Layer; INL, Inner Nuclear Layer; RGCs, Retinal Ganglion Cells; RBCs, Rod Bipolar Cells (×400).

**Figure 5:**
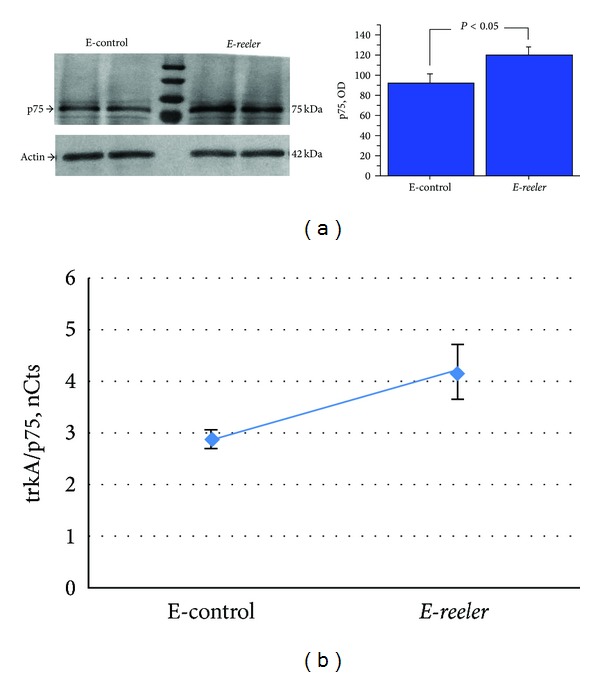
p75^NTR^ expression in *E-reeler* retina. (a) Representative 7.5% SDS-PAGE and relative densitometric analysis (OD values; *P* < 0.05), probed with p75^NTR^ antibodies, showing a significant increase of p75^NTR^ in *E-reeler* protein extracts, as compared to the E-control. (b) Scatter plot showing the correlation with the trkA^NGFR^/p75^NTR^ ratio in the *E-reeler* versus E-control, highlighting a shift towards p75^NTR^ expression (Tau = 0.857; *P* < 0.01).

Given that NGF binds to p75^NTR^, the expression and localization of this glycoprotein were also investigated. In *E-reeler* retinas, p75^NTR^ immunoreactivity increased in RBCs, RGCs, and other INL populating cells, as highlighted by arrows in (Figure 4). Densitometric analysis quantified a 44.7% increase of p75^NTR^ immunoreactivity in the *E-reeler* retina (19587 ± 1916 versus 13539 ± 1368 IntDen, resp., *E-reeler* versus E-control; *P* < 0.05). In line, Western blot analysis coupled to densitometric quantification showed a significant 32.5% increase of p75^NTR^ in *E-reeler* protein extracts (*P* < 0.05; Figure 5(a)). The increased p75^NTR^ protein expression in *E-reeler* retinas was corroborated by real time PCR (1.69_2log⁡_ ratio; 4.29 ± 1.77 versus 5.73 ± 3.22 nCts; *E-reeler* versus E-control; *P* < 0.05). The decrease of RBCs (GFP^+^ cells) did not correlate significantly with the increase of p75^NTR^ immunoreactivity (OD), as detected by Kendall rank analysis between the *E-reeler* and E-control values (Tau = −0.308; *P* > 0.05). Interestingly, the trkA^NGFR^/p75^NTR^ ratio (from nCts values) was decreased in *E-reeler* mice as compared to the E-controls, indicating a shift towards p75^NTR^ expression (Tau = 0.867; *P* < 0.01, Figure 5(b)).

## 4. Discussion

This study was undertaken to verify the NGF-trkA^NGFR^/p75^NTR^ expression in retinas from* E-reeler* mice, a Reelin-deprived model showing severe structural and functional changes in the retina, alongside the severe central nervous system alterations. To date, no data are available on the NGF pathway in Reelin deficient retina.

Retinogenesis is driven by a milieu of soluble factors, synergizing to obtain the final well-organized synaptic circuitry [1, 8]. As observed in several experimental models, Reelin and NGF take part actively during retinogenesis and continue all over adulthood, contributing to retina homeostasis (synaptic plasticity) [1, 3, 7, 8]. Structural and functional changes in the retina have been detected in Reelin deficient mice, showing an altered distribution of RBCs (both cell malpositioning and reduced dendrite density) and impaired synaptic circuitry [1]. RGC apoptosis has been quantified upon chemical NGF deprivation, while the recovery of damaged structures and particularly NGF/trkA^NGFR^-promoted RGC survival were observed upon the intraocular NGF injection, as observed in models of retinal degeneration (ischemia, glaucoma, and diabetes) [11, 26–28].

Since emerging data lead to a possible cross-talk between Reelin and NGF during retinogenesis and tissue remodeling, the well-characterized *reeler*-L7-EGFP model was developed (*E-reeler*) and Reelin-deprived retinas underwent NGF-trkA^NGFR^/p75^NTR^ confocal microscopy and biomolecular characterization [16, 17]. The morphological analysis of *E-reeler* retina at P7-P35 showed structural changes characterized by altered arrangement and significant decrease of axon/dendrite density of residual RBCs (retina degeneration), in line with previous studies [1, 6, 8]. Ataxia and eating and survival difficulties were observed, and since the survival was highly reduced over P27 in the absence of appropriate management, the P21 was definitely selected for these studies. According to the digital analysis and the conventional cell-counting method, significant reductions in RBCs' soma, dendrites, and axons were quantified in *E-reeler* retina. This observation is in line with previous studies indicating that just RBCs represent the primary target of Reelin defect, even though both RBCs and RGCs express/react to Reelin [1, 5]. Since RBCs carry the signal from the rod photoreceptors to the RGCs (visual function), it is reasonable to hypothesize that RBCs defect might interfere with the physiological activity of RGCs in *E-reeler* retina.

The principal finding of this study is the significant increase of NGF all over *E-reeler* retina, mainly localized in the layers populated by RBCs (INL) and RGCs (GCL). Indeed, NGF immunoreactivity was greatly expressed in accessory/glial cells, representing certainly the main NGF source in the damaged neighbourhood [29–32]. The observation of increased NGF protein, not corroborated by the molecular data, might be explained as possible transcriptional/posttranscriptional regulations (differential regulation, stability, and degradation of mRNA). In line with the well-known NGF pleiotropic effects, this NGF increase might be explained as an endogenous compensatory response to Reelin deficiency, either to limit abnormal/impaired cell distribution or counteract undesired apoptosis [12, 26, 33].

The statement that NGF exerts multiple effects depending on the surface (co)receptor appearance strength suggests the potential contribution of trkA^NGFR^ and/or p75^NTR^ in this model. In the retina, p75^NTR^ privileges Müller glial cells while trkA^NGFR^ is mainly expressed by RGCs [30, 34]. While trkA^NGFR^ tasks in neuronal survival, growth, and synaptic modulation are well established, p75^NTR^ ones are still an open debate due to p75^NTR^-trkA^NGFR^ coreceptor activity/complexity [15, 33, 35]. p75^NTR^ mediates a widespread range of cellular functions, depending on the cell-to-cell and/or cell-to-factor milieu as well as the repertoire of surface (co)receptors and can signal independently of trkA^NGFR^ [12, 36, 37]. In the nervous system, p75^NTR^ mediates neuronal survival by facilitating trkA^NGFR^ signal, increasing neuronal axon growth and reducing neuronal cell death [37–42]. As a second finding, a significant increase of p75^NTR^ and a trend towards a decrease of trkA^NGFR^ were observed in *E-reeler* retina, as detected by confocal microscopy, Western blot, and real time PCR analysis. Interestingly, p75^NTR^ was mainly localised in RBCs and cells populating the GCL, either RGCs or accessory ones. In particular for GCL, whether p75^NTR^ is expressed by immature/migrating or mature/positioned RGCs during retinogenesis (or in adult retina) remains to be verified, as accessory cells processed tightly surround RGCs [28, 42, 43]. This p75^NTR^ expression would imply that NGF from RGCs and nearby accessory cells might promote the survival of interneurons (and likewise RBCs) via NGF/p75^NTR^-dependent mechanism, favouring a “rescue response” in an autocrine/paracrine fashion [11, 27, 28, 39]. The contribution of p75^NTR^ in the regulation of RBCs survival has been reported in an experiment of exposure to NGF, BDNF, or Neurotrophin 3 [44]. Therefore, the possibility that p75^NTR^ might exert the herein reported actions through other growth factors cannot be excluded [12, 27].

An attempt for cytoskeleton reorganization might be also prospected for NGF and p75^NTR^ overexpression, since p75^NTR^ is strictly required for appropriate axonal morphology/outgrowth and cell migration [38]. Actin reorganization and cell migration as well as neurite outgrowth are common properties of both NGF and Reelin [1, 4–7, 26, 27]. As reported, the loss of RGCs might be an outcome of the reduced crosstalk with the RBCs (synapses decline) [1]. NGF overexpression in RGCs or accessory/Müller cells might be also viewed as an attempt to stimulate RBC dendrites/arbors elongation, to (re)establish synaptic connections, or to provide a gradient for new dendrites [45–48]. As a support, p75^NTR^ binds both pro/mature forms of NGF and other neurotrophins (NTs, BDNF, NT3, and NT5), takes part in retrograde axonal transport of NTs (as survival or apoptotic factor), and works as a shuttle molecule for BDNF and NT4 (RBC survival NTs) [44, 49]. As reported, NGF pathway contributes to the cytoskeleton reorganization, at least in structural cells as myofibroblasts [50].

By the way, the selective shift toward p75^NTR^ expression does not exclude the potential contribution of trkA^NGFR^ signalling. As reported, p75^NTR^ acts as neuroprotective molecule, and trkA^NGFR^ might work as a death receptor [43, 51]. Upon NGF exposure, both homo- and heterodimerizations of membrane bound trkA^NGFR^ and p75^NTR^ occur on receptive cells, and the ultimate signalling response is the result of some predominant cascade pathways [12, 13]. The dogma “trkA^NGFR^ mediates survival while p75^NTR^ triggers apoptosis” is strictly dependent on the cell type, microenvironment, and trkA^NGFR^/p75^NTR^ surface-expression ratio [12, 15]. In line with our finding, a possible role of trkA^NGFR^/p75^NTR^ ratio in determining the fate of RBCs and RGCs might be prospected, as supported by studies on degeneration rescue [11, 14, 15]. Therefore the p75^NTR^ increase in RGCs and RBCs might also be interpreted as a proapoptotic effect. Studies aimed at verifying/quantifying apoptosis of RGC and/or RBC are actually under investigation.

As widely reported, a milieu of soluble factors synergize to allow for the correct position and functional activity of the RGCs, amacrine cells, RBCs, horizontal cells, Müller glial cells, and rod/cone photoreceptors during retinogenesis [8, 52]. The possibility that upon Reelin deprivation other factors (cytokines and growth and angiogenic factors) might be upregulated to offset or facilitate the entire process cannot be excluded. Beside NGF, several other factors (such as BDNF, NT4, and GDNF) have been reported to increase RGC survival and regeneration [44, 53]. These activities might be direct or indirect as observed for NGF-p75^NTR^-induced TNF-*α* and TGF-*β* activities in developing retina [54].

Any attempt to comprehend the mechanism underlying the pathogenesis of some retinal disorders represents a step forward in ophthalmology field characterized by severe, invalidating, and life-threatening diseases (glaucoma, ischemia, retinopathies, etc.). One of the major challenges of current eye-disease research is to develop models that mimic eyes pathologies, useful tools for studying cell-to-cell and cell-to-mediator mechanisms and providing the basis for novel therapeutic approaches to offset retinal degeneration. Despite recent advances, a clear comprehension of the mechanism underlying retinopathies is still needed and might require the understanding of some “missing” aspects during retinogenesis. For several decades, the *reeler* mutant has been used as a model for studying neurological disorders [1, 16]. In line with recent and the herein presented data, we propose *E-reeler* mice as a good “retinal disease” model to explore the cross-talk between NGF and Reelin [16, 17]. Growing data indicate that NGF provides a potential approach in the treatment of retinopathies, characterized by RGCs death and following optic nerve degeneration [2, 3, 10, 11]. Studies are underway to discriminate p75^NTR^-induced cell rescue or apoptosis and characterize the proNGF/NGF expression. These studies will contribute to better understanding of the relationship between NGF and Reelin in the retina under normal and pathological conditions.

## Figures and Tables

**Table 1:** Primers for genotyping (A) and for real time PCR (B) used in the study.

^a^Gene access number	Sequence (For/Rev)	Amplicon	Annealing conditions
A: genotyping			
^b^Reeler (GM75)	F: 5′-TAA TCT GTC CTC ACT CTG CC-3′	380 bp	55°C, 120 s
^b^Reeler (3W1)	R: 5′-ACA GTT GAC ATA CCT TAA TC-3′	280 bp	
^b^Reeler (3R1)	R: 5′-TGT ATT AAT GTG CAG TGT TG-3′		
^a^GFP 1	F: 5′-CGT AAA CGG CCA CAA GTT CAG-3′	500 bp	65°C, 30 s
^a^GFP 2	R: 5′-ATG CCG TTC TTC TGC TTG TCG-3′		

B: RT-PCR			
^c^GAPDH	S: 5′-GTGGACCTCATGGCCTACAT-3′	100 bp	53°C, 30 s
BC059110	AS: 5′-GTTGGGATAGGGACTCCTCAC-3′		
^c^trkA	S: 5′-AACAACGGCAACTACAC-3′	137 bp	58°C, 25 s
M23102	AS: 5′-CCTGTTTCTCCGTCCAC-3′		
^c^p75^NTR^	F: 5′-GAGGCACCACCGACAACCTC-3′	131 bp	55°C, 25 s
AF187064	R: 5′-TGCTTGCAGCTGTTCCACCT-3′		
^c^NGF	F: 5′-CTGGCCACACTGAGGTGCAT-3′	120 bp	53°C, 30 s
BC011123	R: 5′-TCCTGCAGGGACATTGCTCTC-3′		

Amplification profiles:

^a^1 cycle at 94°C/5 min, 30 cycles including 94°C/1 min, 55°C/2 min, and 72°C/3 min, and a final cycle at 72°C/10 min.

^b^1 cycle at 94°C/5 min, 35 cycles including 94°C/30 sec, 65°C/30 sec, and 72°C/30 sec, and a final cycle at 72°C/10 min.

^c^1 cycle at 95°C/15 min, 47 cycles of denaturation at 95°C/30 sec, annealing at 55–60°C/25 sec (primer's Tm dependent), and elongation at 72°C/30 sec, fluorescence monitoring at 60–90°C, 0.01°C for 0.3 sec, and final incubation at 72°C/5 min. Single melting curves always verified.
